# Differing Definitions of Outpatient Surgery May Influence Study Outcomes Related to ACL Reconstruction

**DOI:** 10.3390/jcm15010227

**Published:** 2025-12-27

**Authors:** Ryan Hoang, Junho Song, Arthur W. Cowman, Timothy Hoang, Alexander Yu, Justin Tiao, Haiyue Jin, Robert L. Parisien

**Affiliations:** 1 School of Medicine, The University of California, Irvine, CA 92697, USA; rjhoang@hs.uci.edu (R.H.); haiyuej1@hs.uci.edu (H.J.); 2 Icahn School of Medicine at Mount Sinai, New York, NY 10029, USAtimothy.hoang@mountsinai.org (T.H.); alexander.yu@mountsinai.org (A.Y.); justin.tiao@mountsinai.org (J.T.); robert.parisien@mountsinai.org (R.L.P.)

**Keywords:** anterior cruciate ligament reconstruction, outpatient, ACS-NSQIP, complications, knee surgery

## Abstract

**Background:** Anterior cruciate ligament reconstruction (ACLR), one of the most frequently performed orthopedic procedures, has experienced rising demand and escalating costs, driving efforts to reduce expenses through shorter hospital stays and an increased shift toward outpatient settings. This study aims to evaluate how differing definitions of “outpatient” surgery influence the interpretation of outcomes following ACLR. **Methods:** ACS-NSQIP was queried for patients undergoing primary ACL reconstruction between 2014 and 2023. Patients ≥ 18 years with CPT code 29888 were included. Patients with missing hospital length of stay (LOS) data or a LOS > 2 days (≥99th percentile) were excluded. Two definitions of “outpatient” surgery were evaluated: hospital-defined outpatient (HDO) and same-day discharge (SDD, LOS = 0). Propensity score matching of baseline demographics and comorbidities was used to compare HDO and SDD cohorts to their respective inpatient counterparts. Primary outcomes analyzed included 30-day readmission, reoperation, and postoperative complications. Univariate and multivariate analyses were performed to compare risks of complications for HDO and SDD cohorts compared to their inpatient counterparts. **Results:** A total of 37,546 patients were included in this study, with 35,334 HDO (94.1%) and 34,801 (92.7%) SDD cases. 1021 (2.9%) of the 35,334 HDO patients had an inpatient hospital stay of at least 1 night. In propensity-matched cohorts, hospital-defined inpatient ACLR was associated with significantly greater risk of 30-day reoperation (odds ratio [OR] 3.167, 95% CI 1.267–7.915, *p* = 0.009) and superficial surgical site infection (SSI) (OR 5.0, 95% CI 1.712–14.604 *p* = 0.001), while HDO ACLR was associated with increased risk of deep vein thrombosis (DVT) (OR 0.333, 95% CI 0.121–0.916, *p* = 0.025). Compared to the propensity-matched SDD cohort, inpatient ACLR was significantly associated with greater rates of 30-day readmission (OR 1.988, 95% CI 1.088–3.630, *p* < 0.001), reoperation (OR 3.222, 95% CI 1.528–6.794, *p* = 0.001), and superficial SSI (OR 3.286, 95% CI 1.412–7.644, *p* = 0.003). **Conclusions:** This study found differences in readmission and deep vein thrombosis between HDO and SDD cohorts when compared to inpatient ACLR. A standardized definition of outpatient surgery should be created to clearly distinguish same-day discharge from other outpatient categories, considering discharge timing and patient monitoring practices.

## 1. Introduction

Anterior cruciate ligament (ACL) is crucial in stabilizing the knee joint, with injuries causing pain and instability that lead to limited physical activity [1]. Recent biomechanical work, including data-driven deep learning models of ligament fatigue behavior, has further highlighted the complexity of ACL stability and failure mechanisms [2]. An ACL tear may permanently impair knee functionality and increase the risk for early osteoarthritis and chronic pain [1]. ACL reconstruction (ACLR) surgery is among the most commonly performed orthopedic procedures in the United States, with cases rising from 86,687 in 1994 to 129,836 in 2006 [1,3]. This growing demand, coupled with rising costs, has led to a national expenditure of up to $17.7 billion annually [4]. As a result, recent efforts have focused on shorter hospital stays to reduce the financial burden associated with ACLR. The existing orthopedic literature generally agrees that outpatient ACLR offers similar or better outcomes compared to inpatient procedures, including complication rates, pain, patient satisfaction, knee function, and strength [5,6,7,8]. Furthermore, outpatient procedures are generally shown to be more cost-effective [9,10,11,12]. As a result, outpatient ACLR rates increased from 57.3% in 1997 to 95.1% in 2006. Nonetheless, variations in the definition of “outpatient” significantly impact how these statistics and outcomes are interpreted.

A key advantage of large national databases, including the American College of Surgeons National Surgical Quality Improvement Program (ACS-NSQIP), is that cases are classified as either outpatient or inpatient, enabling comparisons across different outcome variables. However, an important distinction is that even when a surgery is coded as “outpatient,” patients may be placed under “observation” status and remain in the hospital overnight for one or more days [13]. While both involve receiving care in a hospital, “observation status” is a specific type of outpatient status where a patient is monitored in the hospital to determine if they need to be admitted as an inpatient, meaning they are not officially considered an inpatient even if they stay overnight. On the other hand, “outpatient status” refers to receiving treatment and leaving the hospital on the same day without an inpatient admission [14]. These heterogenous methods of coding “outpatient” create ambiguity between inpatient and outpatient procedures. Many studies have used national databases to compare short-term outcomes of inpatient versus outpatient surgery [15,16,17,18]. Specifically, studies on outpatient ACLR have used varying definitions of outpatient status. These range from patients discharged on the same day to those staying no more than one night or patients coded as “outpatient” in a database [19,20,21,22,23,24,25]. This variability is possible because in NSQIP, the “inpatient” or “outpatient” classification is separate from the hospital length of stay (LOS) variable, and the “inpatient” or “outpatient” variable often does not match with LOS > 0 days or LOS = 0 days [26]. Understanding the extent of this mismatch and clarifying what “outpatient” truly means can significantly influence the way clinicians interpret data from these large national databases and how hospital systems use these findings to guide patient treatment [26].

The aim of this study is to evaluate whether the definition of “outpatient” surgery, hospital-defined outpatient (HDO) versus same-day discharge (SDD), affects 30-day outcomes following ACLR. We hypothesize that differences in these definitions will lead to discrepancies in reported outcome measures. With this study, we hope to raise awareness about potential inconsistencies and to improve the accuracy of data interpretation in ACLR studies.

## 2. Materials and Methods

### 2.1. Database and Patient Population

This is a retrospective study of prospectively collected data from the ACS-NSQIP database. ACS-NSQIP is an outcomes-based registry containing data from more than 700 participating institutions, including academic and private, community and tertiary, and inpatient and outpatient medical centers [27]. Patient demographic information and 30-day postoperative outcomes are collected from the electronic medical record and undergo regular auditing by clinical reviewers to ensure data accuracy and validity [28]. NSQIP is a national database containing publicly available and deidentified patient data. As such, patient consent and Institutional Review Board approval were not required for this study.

Patients who underwent primary ACLR surgery between 2014 and 2023 were identified using Current Procedural Terminology (CPT) code 29888. Exclusion criteria included patients with missing LOS data (variable “TOTHLOS” code-99; N = 17) and patients with LOS at or above the 99th percentile, or greater than 2 days, to ensure patients who were ultimately admitted to the hospital were excluded from analyses (N = 396). Furthermore, the CPT code 29888 ensures patients undergoing revision surgeries were excluded.

### 2.2. Definitions of “Outpatient” Surgery

The first method of classification of “outpatient” surgery was HDO, which was defined using the NSQIP-provided inpatient/outpatient (variable “INOUT” code Outpatient) variable. HDO refers to a distinct variable derived from each individual hospital’s definition of inpatient and outpatient status. The second method of classification of “outpatient” surgery was SDD, which was defined as LOS = 0 (variable “TOTHLOS” code 0). The SDD cohort only includes patients who did not stay overnight in the hospital following surgery. The “inpatient” cohort was defined as either the hospital’s individual definition of inpatient, hospital-defined inpatient (HDI; variable “INOUT” code Inpatient), or as patients with LOS > 0, different-day discharge (DDD; variable “TOTHLOS” code > 0), depending on the comparative group, HDO or SDD, in the statistical analyses. HDO cases were matched with HDI cases, while SDD cases were matched with DDD cases, where LOS > 0.

### 2.3. Studied Variables and Outcomes

Demographic and perioperative variables were collected from ACS-NSQIP. Demographic variables included age, sex, race, ethnicity, and body mass index (BMI). Perioperative variables included operation time, LOS, discharge disposition, and hospital-defined outpatient. Operative time was measured as minutes between initial incision and end of wound closure. LOS was measured as the number of calendar days from the index procedure to discharge. Comorbidities, including diabetes mellitus, chronic obstructive pulmonary disease (COPD), congestive heart failure (CHF), hypertension, smoking status, chronic steroid use, functional dependence, and American Society of Anesthesiologists (ASA) classification, were recorded.

The primary postoperative outcomes analyzed were the incidences of 30-day readmission, reoperation, and morbidity. Individual complications were also recorded, including superficial surgical site infection (SSI), deep SSI, pneumonia, pulmonary embolism (PE), urinary tract infection (UTI), stroke, myocardial infarction, and deep venous thrombosis (DVT).

### 2.4. Statistical Analyses

Demographic and clinical characteristics were compared between HDO and SDD cohorts (Table 1). Frequencies and proportions were recorded for each categorical variable, while medians and interquartile ranges (IQR) were recorded for each continuous variable. To compare outpatient vs. inpatient cohorts based on the different definitions, propensity score matching was utilized to match “HDO” to “HDI” and “SDD” to “DDD.” Propensity score matching was utilized to minimize confounding effects due to differences in baseline patient characteristics [29]. In our analysis, each HDO and SDD case was matched with their respective inpatient counterparts, without replacement, with regard to age, sex, black race, Hispanic ethnicity, BMI, functional dependence, ASA class ≥ 3, diabetes mellitus, smoking status, COPD, CHF, hypertension, and chronic steroid use. Patients were matched 1:1 using nearest-neighbor propensity score matching, and a match tolerance (caliber width) of 0.1 was used to match the HDO and SDD cohorts to their inpatient counterparts. Following matching, baseline characteristics of the post-matched HDO–HDI and SDD–DDD cohorts were summarized with standardized mean differences (SMD) reported for all variables included in the propensity model to gauge post-matching covariate balance achieved through the matching process. Two distinct sets of univariate analyses, with odds ratio (OR) and chi-squared analyses, were performed to calculate the risks of readmission, reoperation, morbidity, and complications for HDO and SDD compared to their inpatient counterparts. Following propensity score matching, multivariable logistic regression was performed for each significant postoperative outcome, adjusting for all covariates used in the matching model (age, male sex, black race, ethnicity, BMI, diabetes, smoking status, functional dependence, COPD, CHF, hypertension, chronic steroid use, and ASA class ≥ 3). Outpatient status (HDO vs. HDI or SDD vs. DDD) was entered as the primary exposure, and adjusted odds ratios with 95% confidence intervals were reported for all models. The level of significance was set at *p* < 0.05. Statistical analyses were performed using SPSS version 28 (IBM, Armonk, NY, USA).

## 3. Results

**Figure 1:**
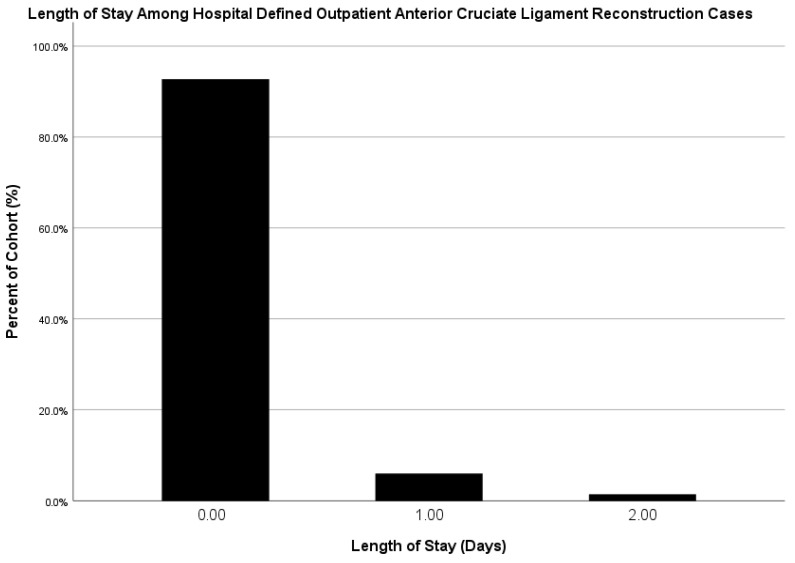
Length of Stay Among Hospital-Defined Outpatient ACL Reconstruction Cases.

**Figure 2:**
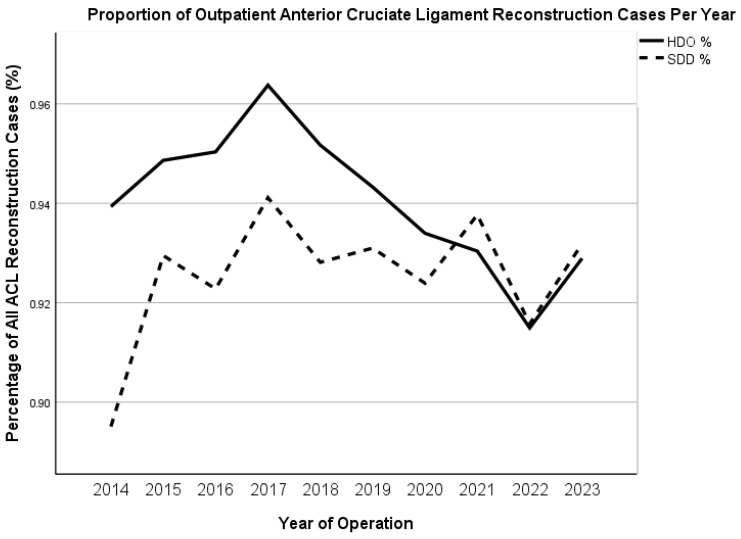
Proportion of Outpatient ACL Reconstruction Procedures by Year for Hospital-Defined Outpatient (HDO) and Same-Day Discharge (SDD).

A total of 37,546 patients were included in this study, with 35,334 HDO (94.1%), 2212 HDI (5.9%), 34,801 SDD (92.7%), and 2745 DDD (7.3%) cases. 1021 of the 34,678 (2.9%) HDO patients had an inpatient LOS of at least 1 night. (Figure 1). Between 2014 and 2023, the annual proportion of HDO ACLRs decreased from 93.9% to 92.9%, while the annual proportion of SDD cases increased from 89.5% to 93.2% over the same period (Figure 2).

The HDO and SDD cohorts have no significant differences in baseline characteristics, including median age, sex, black race, Hispanic ethnicity, and median BMI (all *p* > 0.05). There were no significant differences between cohorts for comorbidities, including functional dependence, ASA class, diabetes, smoking status, COPD, CHF, hypertension, and steroid use. For perioperative variables, the SDD cohort was significantly associated with shorter median operative time (99 min vs. 100 min, *p* = 0.009) compared to the HDO cohort. The SDD cohort consisted of 34,313 HDO (98.6%) cases, while the HDO cohort had a LOS of 0.03 ± 0.19 days (*p* < 0.001; Table 1). Furthermore, HDO vs. HDI and SDD vs. DDD baseline demographics and comorbidities are described in Supplemental Tables S1 and S2.

In the propensity-matched HDO cohort, inpatient ACLR was associated with significantly greater risk of 30-day reoperation (OR 3.167, 95% CI 1.267–7.915, *p* = 0.009) and superficial SSI (OR 5.000, 95% CI 1.712–14.604, *p* = 0.001). However, inpatient ACLR was also associated with a significantly lower risk of DVT (OR 0.333, 95% CI 0.121–0.916, *p* = 0.025) compared to the HDO group (Table 2). In comparison to the propensity-matched SDD cohort, inpatient ACLR was significantly associated with greater rates of 30-day readmission (OR 1.988, 95% CI 1.088–3.630, *p* = 0.022), reoperation (OR 3.222, 95% CI 1.528–6.794, *p* < 0.001), and superficial SSI (OR 3.286, 95% CI 1.412–7.644, *p* = 0.003; Table 3).

**Figure 3:**
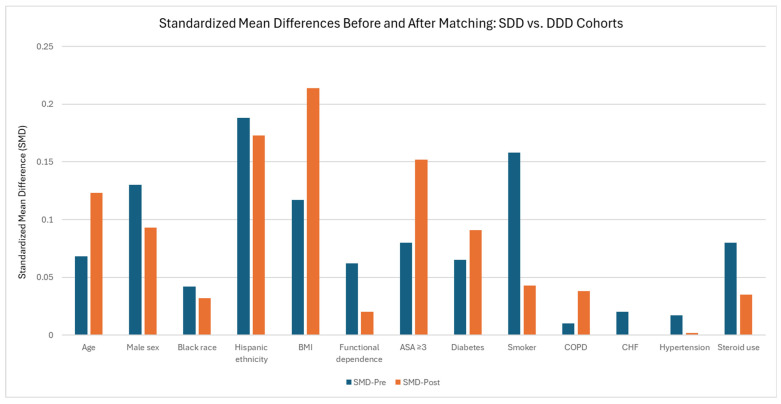
Changes in Standardized Mean Difference of the Same-Day Discharge (SDD) Cohort vs. Different-Day Discharge (DDD) Cohort before and after Propensity Score Matching.

After propensity score matching, 2125 HDO cases were matched to 2125 HDI cases, resulting in the removal of 33,209 patients from the HDO cohort and 87 patients from the HDI cohort (Table 4). Baseline covariates demonstrated generally acceptable balance, with most standardized mean differences < 0.10. However, modest residual imbalance persisted for male sex (SMD 0.235) and Hispanic ethnicity (SMD 0.319). Median age was slightly higher in the HDO cohort (30 vs. 28 years; SMD 0.228), while BMI was similar between groups (SMD 0.071). The prevalence of comorbidities remained low across both cohorts, with all SMDs < 0.13. Differences in SMDs before and after matching in the HDO-HDI analyses are summarized in Figure 3 and Supplemental Table S3.

Perioperative characteristics differed significantly between groups. HDO patients had shorter operative times (median 91 vs. 116 min, *p* < 0.001) and were all discharged on the day of surgery (100% vs. 0%, *p* < 0.001), with a correspondingly shorter median LOS (0 vs. 1 days, *p* < 0.001). Regarding postoperative outcomes, HDO patients demonstrated lower reoperation rates (0.3% vs. 0.9%, *p* = 0.009) and lower rates of superficial SSI (0.2% vs. 0.9%, *p* < 0.001). Rates of readmission, deep SSI, pneumonia, PE, UTI, and mortality were low and statistically comparable between groups. DVT was more common in the HDO cohort (0.7% vs. 0.2%, *p* = 0.025; Table 4).

**Figure 4:**
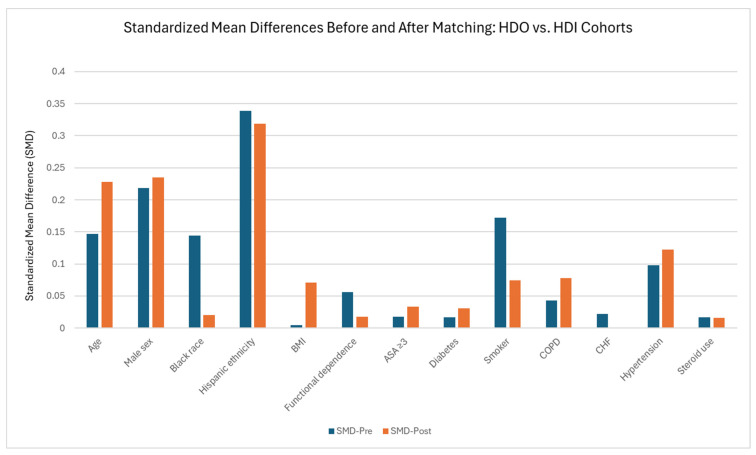
Changes in Standardized Mean Difference of the Hospital-Defined Outpatient (HDO) Cohort vs. Hospital-Defined Inpatient (HDI) Cohort before and after Propensity Score Matching.

Following matching, 2670 SDD patients were matched to 2670 DDD patients, resulting in the exclusion of 32,131 SDD patients and 75 DDD patients (Table 5). Baseline characteristics demonstrated good covariate balance, with most standardized mean differences < 0.10, although mild residual imbalance persisted for Hispanic ethnicity (SMD 0.173), BMI (SMD 0.214), and ASA class ≥ 3 (SMD 0.152). Age (SMD 0.123) and sex (SMD 0.093) were also slightly different between groups but remained within acceptable limits. Comorbidities were similarly distributed across cohorts, with all SMDs ≤ 0.15. Differences in SMD before and after matching in the SDD–DDD analyses are summarized in Figure 4 and Supplemental Table S3.

SDD patients had significantly shorter median operative times (92 vs. 121 min, *p* < 0.001), and almost all were discharged on the day of surgery (98.6% vs. 37.7%, *p* < 0.001), resulting in a markedly shorter median LOS (0 vs. 1 day, *p* < 0.001). Postoperative outcomes favored the SDD cohort, which demonstrated lower readmission (0.7% vs. 1.3%, *p* = 0.022) and reoperation rates (0.3% vs. 1.1%, *p* = 0.001). The incidence of superficial SSI was also lower among SDD patients (0.3% vs. 0.9%, *p* = 0.003). Deep SSI, PE, UTI, pneumonia, DVT, and mortality remained infrequent and statistically similar between groups (Table 5).

Multivariate logistic regression demonstrated that among HDO vs. HDI patients, HDO status was independently associated with reduced odds of reoperation (adjusted odds ratio [aOR] 0.220, 95% CI 0.080–0.605, *p* = 0.003) and superficial SSI (aOR 0.246, 95% CI 0.083–0.730, *p* = 0.012). However, readmission showed no significant association (*p* = 0.104), and the association with DVT demonstrated a notable trend toward significance (aOR 2.756, 95% CI 0.980–7.750, *p* = 0.055). Similarly, within the SDD vs. DDD cohort, SDD status was independently associated with lower odds of reoperation (aOR 0.301, 95% CI 0.141–0.645, *p* = 0.002) and superficial SSI (aOR 0.312, 95% CI 0.131–0.741, *p* = 0.008). Readmission demonstrated a trend toward significance favoring SDD (aOR 0.550, *p* = 0.058), while DVT showed no independent association (*p* = 0.411; Table 6).

## 4. Discussion

This study evaluated how differing definitions of “outpatient” status—HDO and SDD—influence outcomes in ACLR surgery. Among patients analyzed, 94.1% were categorized as HDO, while 92.7% were classified as SDD. Notably, 2.9% of the HDO cohort stayed at least one night in the hospital, underscoring a misalignment between the designated outpatient status and actual discharge practices. These results emphasize the need for clearer, standardized definitions of outpatient surgery to improve data accuracy and outcome reporting.

The proportion of outpatient ACLR procedures remained high from 2014 to 2023, with a slight decrease in annual HDO cases (93.9% to 92.9%) and a marginal rise in SDD cases (89.5% to 93.2%). This shift mirrors a broader movement toward outpatient orthopedic surgeries. For example, Mall et al. reported a significant increase in ACLR cases in the United States, rising from 86,687 in 1994 to 129,836 in 2006, with the proportion performed in outpatient settings increasing from 43% to 95% [30]. These findings align with the general transition toward outpatient ACLR surgeries, consistent with broader trends documented in the literature [1,3,30].

Recent studies have shown that outpatient ACLR offers several advantages, some of which were brought on by the advancements of minimally invasive techniques [30,31]. Outpatient ACLR surgeries may also reduce overall healthcare costs, including facility fees and hospital stay costs [32]. Furthermore, studies have demonstrated that outpatient ACLR patients report higher satisfaction rates and comparable clinical outcomes to those treated in conventional hospitalization settings. For instance, Lunebourg et al. found that patients operated on in an outpatient setting had significantly better satisfaction and similar improvements compared to those in conventional hospitalization [33]. Long-term follow-up studies have shown similar findings, demonstrating improvements in outcomes and high rates of return to sport, with most patients reporting stable knee function and satisfaction years after surgery [34,35].

Our study found no significant differences in baseline characteristics, including age, sex, BMI, or comorbidities, between the HDO and SDD cohorts, suggesting both groups represent comparable patient populations. Regarding perioperative variables, the SDD cohort had a slightly shorter median operative time (99 min vs. 100 min, *p* = 0.009), a statistically significant finding with minimal clinical relevance (Table 1).

Notably, the univariate analysis on the propensity score matched groups revealed that inpatient ACLR is associated with significantly higher risks of adverse outcomes, including 30-day readmission and superficial SSI, compared to both HDO and SDD cohorts. These results highlight the safety and efficacy of outpatient ACLR pathways, particularly SDD, which also demonstrated a significantly reduced risk of reoperation within 30 days of surgery. Furthermore, multivariate analysis confirmed reoperation and readmission to be associated with significantly reduced odds of reoperation and superficial SSI, with readmission trending towards significance in the SDD vs. DDD analysis. These results demonstrate that clinicians can confidently adopt both HDO and SDD as suitable outpatient options for patients with similar demographic and clinical profiles, provided appropriate patient selection criteria are applied.

This study does emphasize that there are discrepancies between the definition of outpatient care and hospital LOS post-procedure for ACLR. We found that 2.9% of the HDO cohort stayed at least one night in the hospital, differing from SDD. Although the patient demographics and perioperative characteristics between the two cohorts were relatively similar, we found differences when comparing outcomes to inpatient ACLR patients. For example, while the propensity score-matched groups demonstrated similar odds of reoperation, the HDI cohort had greater odds of superficial SSI in comparison to the DDD inpatient cohort when compared against their corresponding outpatient counterparts (OR 5.000 vs. 3.286, respectively). Additionally, the outpatient cohort in the HDI-HDO analysis exemplified significantly greater odds of DVT, which may reflect heterogeneity within the HDO designation, as patients classified as “outpatient” can remain hospitalized for several days, potentially experiencing reduced mobility or inconsistent chemoprophylaxis (Table 2). In contrast, the inpatient cohort in the DDD-SDD comparison was associated with significantly greater odds of 30-day readmission following ACLR (Table 3). This likely reflects greater perioperative complexity of early postoperative concerns prompting overnight admission. Thus, if selection criteria for outpatient ACLR were expanded based on risk data that included patients with extended hospital stays, it could inadvertently lead to overly restrictive patient selection for outpatient procedures. While both HDO and SDD denote outpatient procedures, the HDO cohort included patients with a LOS of up to five days before exclusion criteria were applied. If not accounted for, these patients may introduce heterogeneity and potentially impact results. This complicates direct comparisons with SDD cohorts and has been shown to have an effect on outcomes after orthopedic procedures [26,36,37]. For instance, Gordon et al. found that longer operative times, usage of general anesthesia, and non-home discharges had a stronger association with HDO designation when compared to SDD for outpatient total hip arthroplasty [36].

Multivariable regression further clarified these relationships by adjusting for residual imbalances after propensity matching. In the HDO–HDI comparison, HDO status remained independently protective against both reoperation and superficial SSI, whereas the association with DVT demonstrated a borderline trend toward significance, suggesting that prolonged LOS among some HDO-classified patients may influence thromboembolic risk. In the SDD–DDD comparison, SDD status continued to show significantly lower adjusted odds of reoperation and superficial SSI, and a near-significant reduction in readmission risk, reinforcing the clinical distinction between true same-day discharge pathways and hospital-defined outpatient care (Table 6). Collectively, these adjusted analyses underscore that definitional heterogeneity within the HDO designation influences risk estimation and that outcome trends differ meaningfully between HDO- and SDD-based outpatient frameworks.

Additionally, the distinction between inpatient and outpatient ACLR is often blurred by the use of “observation” status, where patients are monitored overnight without formal admission. This practice can lead to classification inconsistencies, making it challenging to interpret results in large national database studies. These findings highlight the necessity of a standardized definition of outpatient surgery that incorporates both LOS and discharge status to ensure consistency across studies and datasets. To address these challenges, we propose developing a universally accepted definition of outpatient surgery. This definition should clearly distinguish same-day discharge from other outpatient categories, considering both discharge timing and patient monitoring practices. Standardization will enhance the reliability of national database studies, enable meaningful comparisons across research, and support evidence-based clinical decision-making. Future research should validate these findings in other surgical contexts and investigate the long-term impacts of outpatient ACLR on patient satisfaction, functional recovery, and healthcare costs.

### Limitations

This study has several limitations. First, the ACS-NSQIP database only captures postoperative data for 30 days, which may not fully capture a patient’s long-term recovery, as any healthcare utilization after this 30-day postoperative period is not included. Additionally, LOS is reported in days rather than hours in this database, limiting the precision for patients whose LOS = 0. In this study, patients with LOS = 0 were considered to have been discharged on the same day as surgery, while those with LOS > 0 were considered to have stayed at least one night in the hospital. The database also does not specify which LOS classification patients placed under a 23 h observation period are classified under and is likely hospital-specific, as this database is composed of hospital-reported data. Since the majority of patients had an LOS of less than two days, more detailed data could offer valuable insights into immediate postoperative recovery patterns amongst these groups. Similarly, because ‘hospital-defined outpatient’ status is based on each institution’s internal billing and classification practices, this variable likely reflects heterogeneous definitions across centers. Outcomes were analyzed within a matched framework to mitigate its potential impact. Furthermore, this study is also limited by unmeasured confounding variables not captured within the NSQIP database, including surgical technique (e.g., graft type), surgeon volume, and institution type, all of which may meaningfully influence perioperative outcomes. There is also the potential issue of miscoding within the NSQIP database, as with any database, but this database has been shown to have high interrater reliability, with a disagreement of around 2%. Another limitation of this study is that the propensity score matching did not fully eliminate imbalance across all covariates; several variables retained SMD values > 0.10, including BMI (HDO–HDI and SDD–DDD), ASA class ≥ 3 (SDD–DDD), Hispanic ethnicity (HDO–HDI), age (SDD–DDD), and smoking status (both cohorts), which may introduce residual confounding (Supplemental Table S3). Finally, as this was a retrospective study, a statistical power analysis was not conducted. Instead, confidence intervals were provided, acknowledging the limitations and potential pitfalls of post hoc power analyses [38,39].

## 5. Conclusions

This study found differences in reoperation and DVT between HDO and SDD cohorts when compared to inpatient ACLR, emphasizing the importance of standardization of the definition of “outpatient” when referring to ACLR. Patients that are defined as outpatients by the hospital may have a LOS of 1 day or more in the hospital, while using the SDD definition ensures that LOS is 0 days. As such, a standardized definition of outpatient surgery should be created, and this definition should clearly distinguish same-day discharge from other outpatient categories, considering both discharge timing and patient monitoring practices.

## Figures and Tables

**Table 1:** Comparison of Baseline Characteristics in ACL Reconstruction Patients in Hospital-Defined Outpatient and Same-Day Discharge Cohorts.

	HDO		SDD		
Characteristic	Number	Percent	Number	Percent	*p*-Value
Total Cases	35,334		34,801		
Median Age (IQR)	29 (15)		29 (15)		0.687
Male Sex	22,320	63.2%	22,035	63.3%	0.683
Black Race	3736	10.6%	3629	10.4%	0.530
Hispanic Ethnicity	4005	11.3%	3902	11.2%	0.608
Median BMI (IQR)	27.5 (6.8)		27.5 (6.6)		0.220
Comorbidities					
Functional dependence	42	0.1%	39	0.1%	0.791
ASA class ≥ 3	1911	5.4%	1825	5.2%	0.335
Diabetes mellitus	488	1.4%	463	1.3%	0.562
Smoker	5137	14.5%	5042	14.5%	0.850
COPD	68	0.2%	65	0.2%	0.863
Congestive heart failure	7	0.0%	7	0.0%	0.977
Hypertension	1989	5.6%	1907	5.5%	0.388
Steroid use	165	0.5%	159	0.5%	0.844
**Perioperative Variables**					
Median operative time (IQR)	100 (60)		99 (58)		**0.009**
Non-home discharge	100	0.3%	94	0.3%	0.745
Hospital defined outpatient	35,334	100.0%	34,313	98.6%	**<0.001**
Median LOS (IQR)	0 (0)		0 (0)		**<0.001**

Bold indicates statistical significance (*p* < 0.05). HDO, hospital-defined outpatient. SDD, same-day discharge. IQR, interquartile range. BMI, body mass index. COPD, chronic obstructive pulmonary disease. ASA, American Society of Anesthesiologists.

**Table 2:** Risk of 30-Day Adverse Events Following Inpatient ACL Reconstruction in Hospital-Defined Inpatient Cases Compared to Propensity Matched Hospital-Defined Outpatient Cohort.

Outcome	Odds Ratio	95% Confidence Interval	*p*-Value
Readmission	1.745	0.891–3.418	0.100
Reoperation	3.167	1.267–7.915	**0.009**
**Individual Complications**			
Superficial SSI	5.000	1.712–14.604	**0.001**
Deep SSI	0.667	0.112–3.986	0.655
Pneumonia	-	-	-
Pulmonary Embolism	2.500	0.486–12.871	0.256
Urinary Tract Infection	1.500	0.251–8.968	0.655
Deep Venous Thrombosis	0.333	0.121–0.916	**0.025**

Bold indicates statistical significance (*p* < 0.05). SSI, surgical site infection—could not be calculated.

**Table 3:** Risk of 30-Day Adverse Events Following ACL Reconstruction in Patients with Length of Stay > 0 Compared to Propensity Matched Same-Day Discharge Cohort.

Outcome	Odds Ratio	95% Confidence Interval	*p*-Value
Readmission	1.988	1.088–3.630	**0.022**
Reoperation	3.222	1.528–6.794	**<0.001**
**Individual Complications**			
Superficial SSI	3.286	1.412–7.644	**0.003**
Deep SSI	3.000	0.606–14.850	0.157
Pneumonia	1.000	0.998–1.000	0.157
Pulmonary Embolism	3.000	0.606–14.85	1.000
Urinary Tract Infection	2.000	0.367–10.910	0.414
Deep Venous Thrombosis	0.833	0.421–1.650	0.600

Bold indicates statistical significance (*p* < 0.05). SSI, surgical site infection.

**Table 4:** Propensity Score–Matched Comparison of Baseline Characteristics and Outcomes Between Hospital-Defined Inpatient and Hospital-Defined Outpatient Cohorts Following ACL Reconstruction.

	HDI		HDO		
Characteristic	Number	Percent	Number	Percent	SMD
Total Cases	2125		2125		
Median Age (IQR)	28 (12)		30 (16.5)		0.228
Male Sex	1561	73.5%	1330	62.6%	0.235
Black Race	144	6.8%	155	7.3%	0.020
Hispanic Ethnicity	61	2.9%	230	10.8%	0.319
Median BMI (IQR)	27.6 (6.3)		26.9 (6.6)		0.071
Comorbidities					
Functional dependence	7	0.3%	5	0.2%	0.018
ASA class ≥ 3	109	5.1%	94	4.4%	0.033
Diabetes mellitus	35	1.6%	27	1.3%	0.031
Smoker	459	21.6%	396	18.6%	0.074
COPD	1	0.0%	9	0.4%	0.078
Congestive heart failure	0	0.0%	0	0	-
Hypertension	74	3.5%	129	6.1%	0.122
Steroid use	8	0.4%	6	0.3%	0.016
**Perioperative Variables**					***p*** **-value**
Median operative time (IQR)	116 (63)		91 (51)		**<0.001**
Non-home discharge	10	0.5%	6	0.3%	0.316
Hospital defined outpatient	0	0.0%	2125	100%	**<0.001**
Median LOS (IQR)	1 (0)	0 (0)			**<0.001**
Outcomes					
Readmission	24	1.2%	13	0.7%	**<0.001**
Reoperation	19	0.9%	6	0.3%	**0.009**
Mortality	0	0.0%	0	0.0%	**-**
Individual Outcomes					
Superficial SSI	20	0.9%	4	0.2%	**<0.001**
Deep SSI	2	0.1%	3	0.1%	0.655
Pneumonia	0	0.0%	0	0.0%	-
PE	5	0.2%	2	0.1%	0.256
UTI	3	0.1%	2	0.1%	0.655
DVT	5	0.2%	15	0.7%	**0.025**

Bold indicates statistical significance (*p* < 0.05). HDI, hospital-defined inpatient. HDO, hospital-defined outpatient. SMD, standardized mean difference. IQR, interquartile range. BMI, body mass index. COPD, chronic obstructive pulmonary disease. ASA, American Society of Anesthesiologists. SSI, surgical site infection. PE, pulmonary embolism. DVT, deep vein thrombosis. -, could not be calculated.

**Table 5:** Propensity Score-Matched Comparison of Baseline Characteristics and Outcomes Between Different-Day Discharge and Same-Day Discharge Cohorts Following ACL Reconstruction.

	DDD		SDD		
Characteristic	Number	Percent	Number	Percent	SMD
Total Cases	2670		2670		
Median Age (IQR)	28 (14)		30 (15)		0.123
Male Sex	1855	69.5%	1739	65.1%	0.093
Black Race	249	9.3%	225	8.4%	0.032
Hispanic Ethnicity	163	6.1%	291	10.9%	0.173
Median BMI (IQR)	28.1 (7.2)		26.9 (6.2)		0.214
Comorbidities					
Functional dependence	10	0.4%	7	0.3%	0.020
ASA class ≥ 3	196	7.3%	103	3.9%	0.152
Diabetes mellitus	60	2.2%	29	1.1%	0.091
Smoker	554	20.7%	508	19.0%	0.043
COPD	4	0.1%	9	0.3%	0.038
Congestive heart failure	0	0.0%	0	0	-
Hypertension	158	5.9%	159	6.0%	0.002
Steroid use	14	0.5%	8	0.3%	0.035
**Perioperative Variables**					***p*** **-value**
Median operative time (IQR)	121 (74)		92 (52)		**<0.001**
Non-home discharge	16	0.6%	6	0.2%	**0.033**
Hospital defined outpatient	1006	37.7%	2633	98.6%	**<0.001**
Median LOS (IQR)	1 (0)		0 (0)		**<0.001**
Outcomes					
Readmission	35	1.3%	15	0.7%	**<0.022**
Reoperation	29	1.1%	9	0.3%	**0.001**
Mortality	0	0.0%	0	0.0%	**-**
Individual Outcomes					
Superficial SSI	23	0.9%	7	0.3%	**0.003**
Deep SSI	6	0.2%	2	0.1%	0.157
Pneumonia	2	0.1%	0	0.0%	0.157
PE	6	0.2%	2	0.1%	0.157
UTI	4	0.1%	2	0.1%	0.414
DVT	15	0.6%	18	0.7%	0.600

Bold indicates statistical significance (*p* < 0.05). DDD, different-day discharge. SDD, same-day discharge. SMD, standardized mean difference. IQR, interquartile range. BMI, body mass index. COPD, chronic obstructive pulmonary disease. ASA, American Society of Anesthesiologists. SSI, surgical site infection. PE, pulmonary embolism. DVT, deep vein thrombosis. -, could not be calculated.

**Table 6:** Multivariable Logistic Regression of risk of 30-Day Adverse Events Following ACL Reconstruction in Propensity-Matched Cohorts.

Outcome	Adjusted OR	95% Confidence Interval	*p*-Value
**HDO vs. HDI**			
Readmission	0.557	0.275–1.128	0.104
Reoperation	0.220	0.080–0.605	**0.003**
Superficial SSI	0.246	0.083–0.730	**0.012**
DVT	2.756	0.980–7.750	0.055
**SDD vs. DDD**			
Readmission	0.550	0.296–1.021	0.058
Reoperation	0.301	0.141–0.645	**0.002**
Superficial SSI	0.312	0.131–0.741	**0.008**
DVT	1.343	0.665–2.711	0.411

Bold indicates statistical significance (*p* < 0.05). SSI, surgical site infection. HDO, hospital-defined outpatient. HDI, hospital-defined inpatient. DVT, deep vein thrombosis. SDD, same-day discharge. DDD, different-day discharge.

## Data Availability

Data used for this study is available upon request and was obtained through the National Surgery Quality Improvement Program database.

## Supplementary Materials

The following supporting information can be downloaded at: https://www.mdpi.com/article/10.3390/jcm15010227/s1, Table S1: Pre-match HDI vs. HDO cohort characteristics and standardized mean differences; Table S2: Pre-match DDD vs. SDD cohort characteristics and standardized mean differences; Table S3: Difference in standardized mean difference pre- and post-propensity score matching.

## Abbreviations

ACLR	Anterior Cruciate Ligament Reconstruction
ACS-NSQIP	American College of Surgeons National Surgical Quality Improvement Program
LOS	Length of Stay
HDO	Hospital-defined Outpatient
HDI	Hospital-defined Inpatient
SDD	Same Day Discharge
DDD	Different Day-Discharge
ACL	Anterior Cruciate Ligament
SMD	Standardized Mean Difference
CPT	Current Procedural Terminology
BMI	Body Mass Index
COPD	Chronic Obstructive Pulmonary Disease
ASA	American Society of Anesthesiologists
SSI	Surgical Site Infection
OR	Odds Ratio
aOR	Adjusted Odds Ratio
NSQIP	National Surgical Quality Improvement Program
