# Supplementary material for: Differing Definitions of Outpatient Surgery May Influence Study Outcomes Related to ACL Reconstruction
Source: J Clin Med. 2025 Dec 27;15(1):227. doi: 10.3390/jcm15010227 (PMC12787227; doi:10.3390/jcm15010227)
Supplement: Supplementary file 1 [file jcm-15-00227-s001.zip › jcm-3948529-supplementary.pdf]

Supplemental materials:

Table S1: Pre-match HDI vs. HDO cohort characteristics and standardized mean differences.

	HDI		HDO		
**Characteristic**	**Number**	**Percent**	**Number**	**Percent**	**SMD**
**Total Cases**	2212		35,334		
Mean Age (SD)	28 (12)		29 (15)		0.147
Male Sex	1621	73.3%	22,320	63.2%	0.218
Black Race	145	6.6%	3736	10.6%	0.144
Hispanic Ethnicity	61	2.8%%	4005	11.3%	0.339
Median BMI (IQR)	27.6 (6.4)		27.5 (6.7)		0.005
**Comorbidities**					
Functional dependence	9	0.4%	42	0.1%	0.056
ASA class ≥3	110	5.0%	1911	5.4%	0.018
Diabetes mellitus	35	1.6%	488	1.4%	0.017
Smoker	467	21.1%	5137	14.5%	0.172
COPD	1	0.0%	68	0.2%	0.043
Congestive heart failure	0	0.0%	7	0.0%	0.022
Hypertension	79	3.6%	1989	5.6%	0.098
Steroid use	8	0.4%	165	0.5%	0.017

HDI, hospital-defined inpatient. HDO, hospital defined outpatient. IQR, interquartile range. BMI, body mass index. COPD, chronic obstructive pulmonary disease. ASA, American Society of Anesthesiologists. SMD, standardized mean difference.

Table S2: Pre-match DDD vs. SDD cohort characteristics and standardized mean differences

	DDD		SDD		
**Characteristic**	**Number**	**Percent**	**Number**	**Percent**	**SMD**
**Total Cases**	2745		34,801		
Median Age (IQR)	28 (14)		29 (15)		0.068
Male Sex	1906	69.4%	22,035	63.3%	0.130
Black Race	252	9.2%	3629	10.4%	0.042
Hispanic Ethnicity	164	6.0%	3902	11.2%	0.188
Median BMI (IQR)	28.1 (7.2)		27.5 (6.6)		0.117
**Comorbidities**					
Functional dependence	12	0.4%	39	0.1%	0.062
ASA class ≥3	196	7.1%	1825	5.3%	0.08
Diabetes mellitus	60	2.2%	463	1.3%	0.065
Smoker	562	20.1%	5042	14.5%	0.158
COPD	4	0.0%	65	0.2%	0.010

Congestive heart failure	0	0.0%	7	0.0%	0.020
Hypertension	161	5.9%	1907	5.5%	0.017
Steroid use	14	0.5%	159	0.5%	0.08

DDD, different-day discharge. SDD, same-day discharge. IQR, interquartile range. BMI, body mass index. COPD, chronic obstructive pulmonary disease. ASA, American Society of Anesthesiologists. SMD, standardized mean difference.

Table S3: Difference in standardized mean difference pre- and post-propensity score matching

	HDI-HDO			DDD-SDD		
**Variable**	Pre-Match	Post-Match	**ΔSMD**	Pre-Match	Post-Match	**ΔSMD**
Median Age (IQR)	0.147	0.228	-0.081	0.068	0.123	-0.055
Male Sex	0.218	0.235	-0.017	0.13	0.093	0.037
Black Race	0.144	0.02	0.124	0.042	0.032	0.01
Hispanic Ethnicity	0.339	0.319	0.02	0.188	0.173	0.015
Median BMI (IQR)	0.005	0.071	-0.066	0.117	0.214	-0.097
**Comorbidities**						
Functional dependence	0.056	0.018	0.038	0.062	0.02	0.042
ASA class ≥3	0.018	0.033	-0.015	0.08	0.152	-0.072
Diabetes mellitus	0.017	0.031	-0.014	0.065	0.091	-0.026
Smoker	0.172	0.074	0.098	0.158	0.043	0.115
COPD	0.043	0.078	-0.035	0.01	0.038	-0.028
Congestive heart failure	0.022			0.02		
Hypertension	0.098	0.122	-0.024	0.017	0.002	0.015
Steroid use	0.017	0.016	0.001	0.08	0.035	0.045

HDI, hospital-defined inpatient. HDO, hospital defined outpatient. DDD, different-day discharge. SDD, same-day discharge. IQR, interquartile range. BMI, body mass index. COPD, chronic obstructive pulmonary disease. ASA, American Society of Anesthesiologists. ΔSMD, change in standardized mean difference.
